# Practice of general pediatrics in Saudi Arabia: current status, challenges, and opportunities

**DOI:** 10.1186/s12887-022-03648-w

**Published:** 2022-10-29

**Authors:** Yossef S. Alnasser, Alhanouf F. Alabdali, Rahaf I. Alshabri, Sawsan M. Albatati

**Affiliations:** 1 Pediatric Department, King Saud University Medical City, Riyadh, Saudi Arabia; 2 Children’s Hospital, King Fahad Medical City, Riyadh, Saudi Arabia; 3 King Saud Medical School, Riyadh, Saudi Arabia

**Keywords:** Saudi Arabia, General Pediatrics, Primary care, Community Pediatricians, Pediatric Age

## Abstract

**Background:**

In Saudi Arabia, general pediatrics serves children until they are 14 years old. It has contributed to improving the health of Saudi children.

**Method:**

This study adopted a qualitative method and recruited pediatric physicians to investigate status, successes, challenges, and opportunities. Later, data were analyzed using thematic analysis and hermeneutic phenomenology.

**Results:**

This study attracted 13 pediatric physicians for interviews. All participants appreciated the role of general pediatricians, but the trainees had a negative attitude regarding the general pediatrics specialty. They all agreed on providing primary care for all children and recommended that their first visit should occur earlier. Shortage of pediatricians, lack of community pediatricians, busy clinics, limited Arabic resources, and poor communication skills are significant barriers to children receiving adequate care. The majority of pediatricians favor extending the pediatric age to 18 years old. One pediatrician stated, “Youths between ages 14–18 years are lost, adults and we refuse to care for them…” Additionally, pediatricians have concerns about managing developmental delays and behavioral issues. They believe the current pediatric residency provides many opportunities for a brighter future.

**Conclusion:**

General pediatrics is well established in Saudi Arabia. To continue thriving, we need to address some challenges that pediatricians face and attract more residency graduates. The current pediatric residency programs can provide opportunities to address deficit areas.

## Background

Pediatrics is a specialty of medical science that is concerned with children’s physical, mental, and social health from birth to young adulthood, as defined by the American Academy of Pediatrics (AAP) [1]. It covers a broad spectrum of health services, ranging from preventive health care to diagnosis and treatment of both acquired and genetic illnesses. Furthermore, it ensures children’s and youths’ growth and development for prospering in their society.

Pediatrics is a heterogeneous field of medicine. This heterogeneity extends to include variable ages and developmental stages. Prior to establishing the modern-day field of pediatrics, families, friends, and midwives attended to infants’ and children’s needs. Physicians rarely contributed to this population’s health in the past. As medicine evolved in the 19th and early 20th centuries, there grew an interest in creating a separate field for caring for sick children. The first known hospital in the western world that was devoted entirely to caring for children was the Sick Children’s Hospital, and was established in Paris, France in 1802 [2]. By the 1850s, greater attention was given to the value of specialized training and education to equip future pediatricians [3].

Pediatrics is one of the first specialties founded in Saudi. Arabia [4]. Over the last two decades, it has rapidly become a well-recognized specialty in the young Kingdom, with many subspecialties [5]. Furthermore, it has continued to grow into a successful example for the whole region [6]. In 1981, the Saudi Pediatrics Association (SPA) was instituted, aiming to improve healthcare services provided for children all over the country [7].

This original study investigates general pediatric providers’ and pediatric trainees’ perspectives on the current status of, and how to improve, general pediatrics in Saudi Arabia. It proposes a qualitative methodology through personal interviews. This methodology aims to reach a representative sample of the general pediatric workforce, delivering child healthcare to discover different viewpoints on successes, challenges, and opportunities. This approach can provide new insights on how to address many unvisited territories of child healthcare in the young Kingdom, and it is the first to explore the quality of general pediatrics in the region, as far as we know. Therefore, this study is meant to serve as a bridge between general pediatricians and policymakers in order to overcome challenges in the field and invest more in successes of delivering a high quality child healthcare. Additionally, findings can voice the various new opportunities that might improve the practice of general pediatrics in the Kingdom.

## Methods

### Study design

This study adopted a qualitative approach, using video calls to practice social distancing during the COVID-19 pandemic. Interviews included 10–15 practitioners of general pediatrics in Saudi Arabia. The interviews included experienced pediatricians (each working for longer than ten years), early career pediatricians, and senior pediatric residents. Inclusion criteria were that the participants had to be a Saudi national, practicing physician, having active or educational license, and willing to consent to participate in an one-hour interview. By interviewing doctors at different experience levels and of both genders, we were able to weigh different insights and be more inclusive. All interviews were audio recorded and transcribed later. Facial expression, gestures, body language, tone, and all forms of non-verbal communications were documented to aid in data analysis.

### Interview questions

Interview questions were all open-ended to capture a wide range of insights. The questions were developed based on literature review, practice observations, and experienced pediatricians’ recommendations. To help participants elaborate when needed, certain props were agreed on for each question, and the interviewer was not allowed to use other tools to enrich the discussion. To access our interview questions, please contact the corresponding author.

### Participant selection

We used a snowball sampling method to recruit general pediatricians for interviews. The snowball sampling method is a non-probability sampling technique where existing study subjects will nominate future subjects from their acquaintances for possible interviewing. This method eliminated any potential selection bias from the investigators. The first interviewee was randomly selected from the general pediatric chairmen in Riyadh, Saudi Arabia. For logistical reasons, a residency program at the principal investigator’s institution was selected for inviting pediatric residents to participate. All senior residents’ names were organized in a list and numbered. Senior residents with odd numbers were selected for interviews. Ten senior residents qualified to participate using this method, but only six consented to partake in our study.

**Data analysis**: We adopted a thematic analysis and hermeneutic phenomenology to analyze our data. First, transcriptions of interviews were reviewed using a thematic analysis to identify common denominators. This analysis allowed for themes to emerge to understand the common consensus among participants. Each theme emerged following a six-step analysis: familiarization, coding, reviewing, generating, defining, and writing. Later, adopting hermeneutic phenomenology allowed for illuminating all details and shinning a light on trivial aspects from interviews. Those aspects helped in understanding attitude and participants’ prospective. All analyses were done manually and no computer-based analysis was used.

### Study ethics

The study was IRB approved by King Fahad Medical City, Riyadh, Saudi Arabia, with IRB numbers 20–574. Participants provided consent before partaking in the audio recording and were allowed to leave and withdraw their participation prior to data analysis. All data were kept confidential and used for the purpose of this study only. One interviewer conducted all of the interviews in order to minimize bias. The interviewer was trained to use only designed props and limit facial expressions and interruptions as much as possible.

## Results

### Study participants

The study was able to attract pediatricians from four hospitals in Riyadh, Saudi Arabia. Many pediatricians (a total of five) declined to participate and did not consent to be audio recorded due to the novelty of medical qualitative research in Saudi Arabia. Even residents were hesitant, and four excused themselves from participating. Table 1.

**Table 1:** Study participants? characteristics

Participants’ Characteristics	Number
Participants	13 (100%)
Pediatric consultant (Attending)	7 (54%)
Pediatric Trainee (Senior Resident)	6 (46%)
Experience longer than 10 years	3 (23%)
Male	5 (38%)
Female	8 (62%)

### Value and attitude

All consultants and trainees appreciate general pediatricians’ roles in Saudi society. They understand the value of their care and feel they have a more significant part to play in population growth and the Saudi 2030 vision. A pediatrician stated, “General pediatric is a basic necessity next to education for Saudi society,*”* and another pediatrician said, “General pediatric is the cornerstone of pediatric medicine, and no hospital can function without it.” However, trainees had a negative attitude toward general pediatrics as a specialty. One trainee stated, “It (general pediatrics) is a non-specialized field (laughing), yes, really!”

### Type and time of Care

The majority of participants think that general pediatricians should be considered primary care providers in Saudi Arabia. They believe in their duty to both healthy and sick children. The idea of having a general pediatrician for each child in the Kingdom was a common goal among participants. Still, all pediatricians voiced concerns about the lack of qualified providers to meet public demands. One pediatrician stated, “Saudi public is ready for the idea of a pediatrician for every child, but we do not have enough doctors.” Regarding the current “Well Baby Clinic Module of Care,” all participants raised some concerns. From being overcrowded to the lack of qualified providers, the module is not meeting the Ministry of Health’s designed standard of care. A pediatric resident protested, “Clinic (well-baby clinic) is open for vaccines (only).” The current scheduled first visit at the age of two months was subject to criticism by all participants. They all wished for an earlier first visit, ranging from between two days from discharge to a maximum of the first two weeks of life. The first visit can be used for early detection, breastfeeding support, follow-up on jaundice, and anticipatory guidance for parents, especially first-time parents. All of the pediatricians shared stories of complications of the current delayed first visit. One pediatrician had a child developing kernicterus because of jaundice and delayed presentation to pediatric care.

### Age

The majority of consultants suggested expanding the age of a pediatric patient beyond 14, which is the current cut-off age for pediatric care in most hospitals in Saudi Arabia. Five of the six consultants proposed that 18 be the new cut-off age, with one pediatrician asking to push it up to 21. Only one consultant was content with the current cut-off age. However, the consultant would not mind expanding the pediatric age limit to 18 if the rules of pediatric practice changed in Saudi Arabia. In contrast, residents were reluctant to extend the current age further for all children and suggested customizing the age limit based on needs. A resident declared some concerns about the challenges of caring for adolescents. This was echoed by a pediatric consultant who stated that “Youths between ages 14–18 years are lost, adults and we refuse to take care of them. I think we can provide them care as long as we address some cultural sensitivity and follow Islamic rules.” Furthermore, most consultants doubted the readiness of the current pediatric workforce to provide good care for youths. Nevertheless, a pediatrician announced, “This generation is better than mine, and they will be ready because they have better training.”

### New era, new issues

All of the participants declared some concerns about caring for children with developmental delays and behavioral issues. Some residents asked for more training despite having four weeks of training in child development in the current Saudi pediatric residency curriculum. However, pediatricians think the problem is poor exposure rather than lack of knowledge. One pediatrician blamed the current general pediatric culture of immediately referring every behavioral issue instead of managing it and gaining more experience when he said, “At the least, we have to start treating ADHD as it is common and easy to diagnose. Referral to child development takes up to six months; who will help the child until then?” Furthermore, vaccine hesitancy was identified as a new issue that pediatricians are facing in Saudi Arabia. Another pediatrician expected to deal more with substance use and eating disorders in the near future.

### Challenges

The biggest challenge that all participants recognized is the high public demands and the low number of pediatricians per capita. A pediatrician emphasized that the needs might increase even further with the current Saudi population growth and fertility rates. Furthermore, all of the participants found the time allocated for each visit to be challenging. Another challenge acknowledged was the lack of community pediatricians outside of tertiary and secondary hospitals. All of the pediatricians agreed that separating well-baby clinics from general pediatric practice lowered the quality of care for infants. All of the participants admitted that poor communication skills are one of the existing barriers to improving general pediatric care. Likewise, all of the participants considered it a significant challenge to find Arabic resources to educate the parents. One resident demanded, “Actually, we lack Arabic materials.”

### Opportunities

All participants were proud of the progress that Saudi pediatric training has made through the years. Participating pediatricians asked for improvement in leadership skills and obtaining more procedures in the Saudi pediatric residency program. Similarly, all participants were happy that there was mandatory communication skills training during the Saudi pediatric residency, but asked for further training. Residents want to be involved in designing and improving their training. Another resident asked to make education “less intimidating.” With the higher number of graduates, some public needs can be met, and care can be improved.

Nevertheless, we need to encourage more residency graduates to pursue a career in general pediatrics, as three pediatricians explained. One pediatrician suggested adding a “Billing System” to attract more graduates and encourage better productivity than the current base salary system. Another pediatrician demanded channeling the pediatric residency focus to meet the Saudi public’s needs, especially in addressing autosomal recessive syndromes, complex care, and car safety. On the other hand, all participants had a positive attitude regarding virtual health and thought it can improve access to care, especially for patients from rural areas. Lacking a physical exam might be a downside of this innovative approach as one pediatrician and two residents mentioned.

### Resources

All of the participants identified the American Academy of Pediatrics (AAP) as their primary reference. The Canadian Pediatric Society (CPS) and UpToDate came second in most of the participants’ lists of resources. All of the participants complained about the cost of accessing these references, except for CPS, which is a free educational platform. Two residents recognized NJEM Plus—a continuous self-learning tool from the Saudi Commission for Health Specialties—as a good reference. All of the participants were aware of the Saudi Pediatric Association (SPA). A pediatrician appreciated SPA’s continuous medical education hours while others wished for more. They hoped to see it more involved in building the Saudi pediatric practice guidelines, advocacy, providing Arabic materials, building young leaders, promoting pediatric science, addressing Saudi culture, and being a voice for pediatricians.

## Discussion

Pediatrics pioneers and first-generation child health advocates in the young Kingdom of Saudi Arabia have accomplished a lot in a short period of time. Their accomplishments are measured by considerable improvement in child health and lower mortality rates. Now, only seven children per 1000 die before their fifth birthday compared to 160 children in 1972 [8].

Despite suggestions from study participants to offer general pediatrics as a primary care service, it would be impractical to do so with the limited number of current practicing general pediatricians and the lack of community pediatricians. Integrating general pediatrics with family physician practices and offering pediatric training to family medicine trainees can be temporary alternatives until a higher workforce is available. Successful examples of training family medicine trainees are well established in countries like Canada [9]. Luckily, such training is already in place in some parts of Saudi Arabia. Furthermore, having community pediatricians is crucial and cost-effective in Saudi Arabia with the overutilization of pediatric emergency rooms [10]. A study by Porter B. et al. showed that a pediatric community practice, rather than a tertiary-based general pediatric, can lower a child’s number of emergency visits [11].

Pediatric care may start periconceptionally and proceed from early gestation to early adulthood. The AAP previously released a statement on the age limit for pediatrics in 1988, which was reaffirmed in 2012, and established the upper age limit as 21 years [12]. Alternatively, the CPS defines the upper age limit as 18 years of age. Despite recommendations from the Saudi Health Council to treat children until the age of 16 in pediatrics, the pediatric age limit varies among institutions and wide between 12 and 14 years old. Knowing that nearly 30% of the Saudi population is under 14 years of age obligates a better-accustomed age limit of pediatrics in order to include all adolescents, especially middle and late adolescents (15–18 years old).

Communication skills have been a hot topic of discussion in Saudi pediatric literature. They have always been criticized and deemed deficient [13] [14]. On the bright side, awareness of poor communication skills among trainees and pediatricians has improved, compared to the findings in an earlier report [15]. Additionally, it is promising to see initiatives already in place to improve communication skills during residency training. However, communication skills training should start at earlier stages of medical education in medical schools and be culturally appropriate.

Recent reports documented a high prevalence of pervasive developmental disorders in Saudi Arabia [16]. Additionally, consanguinity has been linked to developmental delay [17]. Consanguinity is a common practice in Saudi Arabia [18]. Dealing with behavioral and developmental issues should not be limited to pediatric development specialists and child psychiatrists. Pediatric residency should prepare future pediatricians to address these issues, and some initiatives have already been implemented.

General pediatric residency training programs in Saudi Arabia have evolved tremendously over the past decade. Pediatric residency training is intended to instill the expertise, skills, and attitudes needed for family-centered healthcare. Likewise, it needs to have a prominent role in meeting complex 21st century health needs and demonstrating an overlap between clinical pediatrics and public health issues. Most importantly, it needs to address public needs while practicing culturally appropriate care. Having giant and well-resourced organizations as references shows an eagerness to learn and follow recent updates among Saudi pediatricians and trainees. However, there is a need for a local organization to address culturally sensitive topics and unique problems affecting Saudi children and youths, like fasting Ramadan for youths with type 1 diabetes or addressing the prevalence of certain metabolic diseases because of consanguinity. Fortunately, there is a well-established SPA, and hopes are high for its future role in generating practice guidelines, advocacy, health literacy, and meeting Saudi children’s needs.

## Conclusion

Saudi general pediatrics is well established and has made considerable contributions to Saudi society. It needs to recruit more residency graduates in order to meet public demands and 21st century needs. The goal is to have a primary care general pediatric service starting with an early first visit in the first few days of life for every child in Saudi Arabia. Pediatricians want to advance the current age limit to include more adolescents. They feel unready to address developmental delay and behavioral issues, and ask for more exposure to such cases. High demands, a low number of qualified physicians, poor communication skills, limited allocated clinic time, unsuccessful well-baby clinic design, and lack of enough community pediatricians are significant challenges for general pediatrics in Saudi Arabia. On the other hand, the current pediatric residency training gives a lot of hope for a brighter future. From training residents on communication skills to addressing developmental delays and managing adolescents, the next generation of general pediatricians will thrive in the field. More importantly, they will continue improving child and youth health in Saudi Arabia and beyond.

## Limitations

The number of participants was limited due to the novelty of qualitative methodological studies in Saudi Arabia. Many pediatricians were not comfortable with this approach and declined to participate. Even pediatric residents expressed dissatisfaction with this method. Additionally, it was hard to arrange one-hour interviews around their busy clinical schedules. All of the participants showed considerable reluctance to record the interviews. Another significant limitation was the inability to recruit pediatricians from other cities, despite all efforts. However, this paper can set foundations for many more studies to help in improving general pediatrics in Saudi Arabia.

RIA: Acquisition of data, analysis and interpretation of data, revising the manuscript, and final approval of the version to be published.

SMA: Substantial contributions to conception and design, drafting the article, revising it, and final approval of the version to be published.

## Data Availability

All of the data generated during and/or analyzed during the current study are available from the corresponding author on reasonable request.
